# Supplementary material for: Factors associated with adherence to antiretroviral therapy among HIV-positive adolescents and young adult patients attending HIV care and treatment clinic at Bombo Hospital in Tanga region-Tanzania
Source: PLoS One. 2025 Jan 3;20(1):e0316188. doi: 10.1371/journal.pone.0316188 (PMC11698315; doi:10.1371/journal.pone.0316188)
Supplement: Attachment — Submitted filename: Review summary of ART adherance.docx [file pone.0316188.s001.docx]

1. **Summary of the research and your overall impression**

When we say "Adherence to antiretroviral therapy among HIV-positive adolescents and young adult patients attending clinic" we are referring to the extent to which HIV-positive adolescents and young adults follow the prescribed treatment regimen for antiretroviral therapy (ART) at the clinic. This includes taking the medication as directed, following the recommended dosages, and adhering to the treatment schedule. Adherence to ART is essential for managing HIV infection and preventing disease progression. It also plays a crucial role in reducing the risk of transmission and improving the overall health outcomes of HIV-positive individuals. The study of adherence to ART among this specific demographic group aims to identify factors that influence adherence and develop interventions to improve treatment compliance.

**1.1 Strengths of the manuscript**

The manuscript's strength lies in its clear and precise writing, sentence coherence, and is focused on critical issues related to antiretroviral therapy (ART). The Authors have sufficiently reviewed relevant literature regarding adherence to ART among adolescents and young adults. The manuscript addresses an important component of adherence to ART among a vulnerable and understudied patient group. Therefore, the study's findings are expected to provide valuable insights into the barriers and facilitators of adherence in this population. This positions the manuscript as a significant contribution to the field of HIV care and management.

**1.2 Weaknesses of the manuscript**

- The Authors did not use any figures to show concepts, procedures, or results.

2. **Discussion of specific areas for improvement**

**2.1 Major issues**

- **Sample size determination**
- You didn't calculate a sample size for your secondary objective (for factors) to check and take the large sample size.
- The prevalence of adherence to ART was 84%. Therefore, you were supposed to use only 2% of margin of error to increase the number of your sample size.

**2.2 Minor issues**

- **In the abstract part**
- Please write the exposing and preventing factors separately. It seems a repetition of the main result. Therefore, mention only the names of variables significantly associated with your dependent variable. (Like A,B,C,and D were significantly associated.)
- Better to write 'moderate food insecurity with (AOR 0.33 (95%CI 0.16-0.66)) and those experiencing ART side effects with AOR 0.61, 95%CI 0.38-0.98) were LESS LIKELY to adhere…..
- X and Y (CI, AOR) were MORE LIKELY to ….
- Please review the punctuation marks
- **Under introduction**
- Please, add more information about the solutions that have been tried in terms of successes and failures with only one paragraph.
- Add gaps in the previous studies from what you reviewed.
- **Sample size determination**
- You did not mention the number of your total population. If your population is less than ten thousand, you could use a correction formula.
- **Under result**
- Be consistent while you write your paper. For instance, the prevalence of adherence to ART in the abstract part and the result part is not consistent (64.7 and 65%).
- **Table 2 Factors associated with adherence to ART**
- A frequency table should be included that shows the percentage of the adherent and non-adherent groups to see and check classification bias. Even it helps to check the Odds ratio manually.
- **Under discussion part**
- Your manuscript says that the differences could be owing to methods of data extraction methods risking under-estimation. It will be better if you clarify the tool differences (extraction methods) between yours and the others in a positive manner.
- **Reference related**
- There are some outdated references like Abrams E, Ammann A, Anderson M, Bernstein L, Baker C. Guidelines for the use of antiretroviral agents in pediatric HIV Infection January 7, 2000. HIV Clin Trials. 2000;1(3):58–99. 30
