# Supplementary material for: Factors associated with adherence to antiretroviral therapy among HIV-positive adolescents and young adult patients attending HIV care and treatment clinic at Bombo Hospital in Tanga region-Tanzania
Source: PLoS One. 2025 Jan 3;20(1):e0316188. doi: 10.1371/journal.pone.0316188 (PMC11698315; doi:10.1371/journal.pone.0316188)
Supplement: Attachment — Submitted filename: Adherance to ART.docx [file pone.0316188.s002.docx]

PLOS ONE

Adherence to antiretroviral therapy among HIV-positive adolescents and young adult patients attending clinic at Bombo Hospital in Tanga region-Tanzania

--Manuscript Draft--

| Manuscript Number: | PONE-D-23-39267 |
| --- | --- |
| Article Type: | Research Article |
| Full Title: | Adherence to antiretroviral therapy among HIV-positive adolescents and young adult patients attending clinic at Bombo Hospital in Tanga region-Tanzania |
| Short Title: | Adherence to antiretroviral therapy among HIV-positive adolescents and young adult patients attending clinic |
| Corresponding Author: | Novatus Apolinary Tesha, PhD Candidate University of Glasgow Muhimbili University of Health and Allied Sciences  Dar es Salaam, TANZANIA, UNITED REPUBLIC OF |
| Keywords: | Adolescents living with HIV, Antiretroviral therapy, Adherence, HIV/AIDS, Tanzania |
| Abstract: | ABSTRACT  Background: Antiretroviral drugs (ART) are potent tools in the fight against HIV, but their effectiveness relies on consistent use. Adherence to ART among Tanzanian adolescents and young adults with HIV/AIDS presents significant challenges, including poor viral load control and heightened health risks. Evidence is lacking in this subpopulation complicating targeted intervention. This study, therefore, aimed to assess adherence levels and identify associated factors among adolescents and young adults receiving ART.  Methodology: This cross-sectional study involved 385 adolescents and young adults living with HIV receiving ART at Bombo Hospital Referral Hospital's Care and Treatment Clinic (CTC). To assess ART adherence, a one-month self-recall medication adherence scale was used, and data on factors affecting adherence were gathered through a structured questionnaire. Stata version 15 was used for analysis through descriptive and logistic regression analyses.  Results: The level of adherence to ART was 64.7% among adolescents and young adults living with HIV. Adolescents and young adults living in households experiencing moderate food insecurity were less likely to adhere to ART (AOR 0.33 (95%CI 0.16- 0.66)) compared to those who were food secure. Those with secondary education were more likely to adhere to ART (AOR 2.31 (95%CI 1.02-5.23)), compared to those without formal education. While participants who consistently obtain their ART at the clinic were more likely to adhere to medication (AOR 4.20, 95%CI 1.29-13.72), those experiencing ART side effects were less likely to adhere to medications (AOR 0.61, 95%CI 0.38-0.98).  Conclusion: More than one-third of adolescents and young adults living with HIV in Tanga, Tanzania were not adherent to ART, surpassing the WHO's 5% threshold. Addressing such unprecedented challenges calls for efforts targeting adolescents and young adults with limited education, from households with food insecurity, and counseling and management of ART side effects. |
| Order of Authors: | Novatus Apolinary Tesha, PhD Candidate University of Glasgow |
|  | Bruno Focus Sunguya, PhD |
|  | Sophia Kamote, MPH |
| Additional Information: |  |
| Question | Response |
| Financial Disclosure  Enter a financial disclosure statement that describes the sources of funding for the work included in this submission. Review the [submission guidelines](http://journals.plos.org/plosone/s/submission-guidelines#loc-financial-disclosure-statement) for detailed | The author(s) received no specific funding for this work. |

| requirements. View published research articles from [PLOS ONE](https://journals.plos.org/plosone/) for specific examples.  This statement is required for submission and will appear in the published article if the submission is accepted. Please make sure it is accurate. |  |
| --- | --- |
| Funded studies  Enter a statement with the following details:   - Initials of the authors who received each award - Grant numbers awarded to each author - The full name of each funder - URL of each funder website - Did the sponsors or funders play any role in the study design, data collection and analysis, decision to publish, or preparation of the manuscript? |  |
| Did you receive funding for this work? |  |
| Competing Interests  Use the instructions below to enter a competing interest statement for this submission. On behalf of all authors, disclose any [competing interests](http://journals.plos.org/plosone/s/competing-interests) that could be perceived to bias this work—acknowledging all financial support and any other relevant financial or non- financial competing interests.  This statement is required for submission and will appear in the published article if the submission is accepted. Please make sure it is accurate and that any funding sources listed in your Funding Information later in the submission form are also declared in your Financial Disclosure statement.  View published research articles from  [PLOS ONE](http://journals.plos.org/plosone/) for specific examples. | The authors have declared that no competing interests exist |

| NO authors have competing interests  Enter: The authors have declared that no competing interests exist.  Authors with competing interests  Enter competing interest details beginning with this statement:  I have read the journal's policy and the authors of this manuscript have the following competing interests: [insert competing interests here] |  |
| --- | --- |
| * typeset |  |
| Ethics Statement  Enter an ethics statement for this submission. This statement is required if the study involved:   - Human participants - Human specimens or tissue - Vertebrate animals or cephalopods - Vertebrate embryos or tissues - Field research   Write "N/A" if the submission does not require an ethics statement.  General guidance is provided below. Consult the [submission guidelines](http://journals.plos.org/plosone/s/submission-guidelines#loc-guidelines-for-specific-study-types) for detailed instructions. Make sure that all information entered here is included in the Methods section of the manuscript. | The study received ethical clearance from the Research and Ethics Committee of MUHAS. – MUHAS – REC-01-2023-1501. After ethical approval, the study team sought authorization to carry out the study from the Tanga Town Council and the Bombo Hospital administration. Before any participant could join the study, they had to go through the consenting process and sign the written informed consent after it was explained to them in clear and plain language. A thumb-printed informed consent was obtained in the case of illiterate participants. Participants under the age of 18 were required to obtain permission from their parents or guardians. The study participants' confidentiality was ensured, and no participants' names were used in the questionnaire |

| Format for specific study types  Human Subject Research (involving human participants and/or tissue)   - Give the name of the institutional review board or ethics committee that approved the study - Include the approval number and/or a statement indicating approval of this research - Indicate the form of consent obtained (written/oral) or the reason that consent was not obtained (e.g. the data were analyzed anonymously)   Animal Research (involving vertebrate animals, embryos or tissues)   - Provide the name of the Institutional Animal   Care and Use Committee (IACUC) or other relevant ethics board that reviewed the study protocol, and indicate whether they approved this research or granted a formal waiver of ethical approval   - Include an approval number if one was obtained - If the study involved non-human primates, add additional details about animal welfare and steps taken to ameliorate suffering - If anesthesia, euthanasia, or any kind of animal sacrifice is part of the study, include briefly which substances and/or methods were applied   Field Research  Include the following details if this study involves the collection of plant, animal, or other materials from a natural setting:   - Field permit number - Name of the institution or relevant body that granted permission |  |
| --- | --- |
|  |  |
| Data Availability  Authors are required to make all data underlying the findings described fully available, without restriction, and from the time of publication. PLOS allows rare exceptions to address legal and ethical concerns. See the [PLOS Data Policy](http://journals.plos.org/plosone/s/data-availability) and [FAQ](http://journals.plos.org/plosone/s/data-availability#loc-faqs-for-data-policy) for detailed information. | No - some restrictions will apply |

| A Data Availability Statement describing where the data can be found is required at submission. Your answers to this question constitute the Data Availability Statement and will be published in the article, if accepted. |  |
| --- | --- |
| Important: Stating ‘data available on request from the author’ is not sufficient. If your data are only available upon request, select ‘No’ for the first question and explain your exceptional situation in the text box. |  |
| Do the authors confirm that all data underlying the findings described in their manuscript are fully available without restriction? |  |
| Describe where the data may be found in full sentences. If you are copying our sample text, replace any instances of XXX with the appropriate details.   - If the data are held or will be held in a public repository, include URLs, accession numbers or DOIs. If this information will only be available after acceptance, indicate this by ticking the box below. For example: All XXX files are available from the XXX database (accession number(s) XXX, XXX.). - If the data are all contained within the manuscript and/or Supporting Information files, enter the following: All relevant data are within the manuscript and its Supporting Information files. - If neither of these applies but you are able to provide details of access elsewhere, with or without limitations, please do so. For example:   Data cannot be shared publicly because of [XXX]. Data are available from the XXX Institutional Data Access / Ethics Committee (contact via XXX) for researchers who meet the criteria for access to confidential data.  The data underlying the results presented in the study are available from (include the name of the third party | Data will be available upon request. The authors will contact the ethical board to abide with the country policy on sharing data internationally. |

| and contact information or URL).   - This text is appropriate if the data are owned by a third party and authors do not have permission to share the data.   * typeset |  |
| --- | --- |
| Additional data availability information: |  |

<https://www.editorialmanager.com/pone/download.aspx?id=34864296&guid=47c5ce80-9c21-4a46-94b0-3d6d3401b0e4&scheme=1>Manuscript	[Click here to access/download;Manuscript;Research Manuscript-Nov 30. 2023.docx](https://www.editorialmanager.com/pone/download.aspx?id=34864296&guid=47c5ce80-9c21-4a46-94b0-3d6d3401b0e4&scheme=1)

# 1 Title: Adherence to antiretroviral therapy among HIV-positive adolescents and young

2 **adult patients attending clinic at Bombo Hospital in Tanga region-Tanzania**
3 Sophia Kamote^1*^, Novatus Tesha^1^ ^2*^, Bruno F. Sunguya^1^
4 ^1^School of Public Health and Social Sciences, Muhimbili University of Health, and Allied
5 Sciences, 9, United Nations Road, Upanga West 11103, Dar es Salaam, Tanzania.
6 ^2^Health Economics and Health Technology Assessment (HEHTA), School of Health and
7 Wellbeing, University of Glasgow, Glasgow, United Kingdom
8 *Corresponding authors: Sophia Kamote ([sophykamote@yahoo.com](mailto:sophykamote@yahoo.com) (SK)) and Novatus Tesha
9 ([n.tesha.1@research.gla.ac.uk](mailto:n.tesha.1@research.gla.ac.uk) (NT))

10

11

12

13

14

15

16

# 17 ABSTRACT

18 **Background:** Antiretroviral drugs (ART) are potent tools in the fight against HIV, but their
19 effectiveness relies on consistent use. Adherence to ART among Tanzanian adolescents and young
20 adults with HIV/AIDS presents significant challenges, including poor viral load control and
21 heightened health risks. Evidence is lacking in this subpopulation complicating targeted
22 intervention. This study, therefore, aimed to assess adherence levels and identify associated factors
23 among adolescents and young adults receiving ART.
24 **Methodology:** This cross-sectional study involved 385 adolescents and young adults living with
25 HIV receiving ART at Bombo Hospital Referral Hospital's Care and Treatment Clinic (CTC). To
26 assess ART adherence, a one-month self-recall medication adherence scale was used, and data on
27 factors affecting adherence were gathered through a structured questionnaire. Stata version 15 was
28 used for analysis through descriptive and logistic regression analyses.
29 **Results:** The level of adherence to ART was 64.7% among adolescents and young adults living
30 with HIV. Adolescents and young adults living in households experiencing moderate food
31 insecurity were less likely to adhere to ART (AOR 0.33 (95%CI 0.16-0.66)) compared to those
32 who were food secure. Those with secondary education were more likely to adhere to ART (AOR
33 2.31 (95%CI 1.02-5.23)), compared to those without formal education. While participants who
34 consistently obtain their ART at the clinic were more likely to adhere to medication (AOR 4.20,
35 95%CI 1.29-13.72), those experiencing ART side effects were less likely to adhere to medications
36 (AOR 0.61, 95%CI 0.38-0.98).
37 **Conclusion:** More than one-third of adolescents and young adults living with HIV in Tanga,
38 Tanzania, were not adherent to ART, surpassing the WHO's 5% threshold. Addressing such
39 unprecedented challenges calls for efforts targeting adolescents and young adults with limited
40 education, from households with food insecurity, and counseling and management of ART side
41 effects.
42 Keywords: *Adolescents living with HIV, Antiretroviral therapy, Adherence, HIV/AIDS,*
43 *Tanzania*

44

45

46

47

48

49

50

51

52

53

54

55

# 56 Introduction

57 HIV/AIDS is a significant and rising public health problem among adolescents, especially in
58 Africa, where it is the leading cause of illness and death in this age group. Globally, 2.1 million
59 adolescents have HIV, with 81% in sub-Saharan Africa (1). In 2020, 410,000 young individuals
60 aged 10–24 years old were newly infected, with 37% being 10–19 years old (2). Tanzania saw
61 about 6,500 new infections in children below 15 years old, and among them, 78% received
62 antiretroviral therapy (ART) (3).
63 ART access has reduced mortality among adolescents with HIV, but adherence remains a
64 challenge. However, only 62.3% of adolescents in sub-Saharan African countries have self-
65 reported being adherent to ART (4). High adherence (over 95%) is crucial for effective viral
66 suppression, preventing drug resistance, and extending life expectancy (5,6). ART offers clinical
67 benefits like viral suppression, disease halt, immune restoration, improved survival rates, reduced
68 illness, and a better quality of life (7,8).
69 Adolescent ART adherence has been associated with demographic, socioeconomic, and behavior-
70 related factors among others (4,9). Such evidence varies with context but remains scant in
71 Tanzania. This study was conducted at Bombo Hospital in Tanga, Tanzania to assess the extent of
72 ART adherence and identify associated factors among the population receiving ART at the
73 hospital.

# 74 Methodology

75 **Study design**
76 This study employed a hospital based cross-sectional design and collected quantitative data for the
77 period of three months to investigate the magnitude and factors associated with adherence among
78 adolescents and young adult clients who received treatment at the Care and Treatment Clinic
79 (CTC) of Bombo Hospital in the Tanga region.

# 80 Study area

81 The study was conducted at Bombo Regional Referral Hospital in Tanga City, Tanzania. HIV is
82 prevalent among 5.4% of adults aged 15-49 years of age, and among 0.8% among those aged 0 to
83 14 years in this region (10). Further evidence suggested a prevalence of 3.68% among those aged
84 10 - 24 years old (11). Moreover, 13.8% of those aged 10 - 24 years have viral load (VL) above
85 50 copies while 6.17% have VL above 1000 copies (11).

# 86 Study populations

87 The study recruited adolescents and young adults who were attending the CTC clinic at Bombo
88 Regional Referral Hospital**.** Adolescents aged 15 - 19 years account for an asymmetrically high
89 number of new HIV infections in Tanzania (12) as do young adults aged 19 - 24 years. The
90 mortality rate for clients living with HIV/AIDS in Tanzania was significantly higher among
91 adolescents and young adults, standing at 3.8 and 2.1 per 100 persons per year (13). Furthermore,
92 despite lower rates of HIV prevalence compared with other age groups, the number of adolescents
93 living with HIV in the country is increasing (14). The study included all adolescents and young
94 adult who are HIV positive clients who had been on ART for 6 months and above and were
95 registered at Bombo CTC during the data collection period, with the exclusion of adolescents and
96 young adults who were too ill to participate.

# 97 Sample size

98 The study included a total of 384 adolescents and young adults who were attending the CTC clinic
99 at Bombo Regional Referral Hospital. The calculation of the sample size used the estimated
100 prevalence of adherence to ART at 84% (15), within a 95% confidence interval and a 5% margin
101 of error.

# 102 Sampling methods

103 The study employed a systematic sampling procedure, where study units were selected from the
104 ART register using a pre-calculated sampling interval. The ART register was used as the sampling
105 frame, and an interval of five was used. For participant recruitment, every fifth registered client
106 was selected to be part of the study.

# 107 Variables and measures

108 Adherence to ART was the dependent variable. It was assessed using the internationally validated
109 one-month self-recall medication adherence scale, which included eight items with a binary
110 response (16). An overall higher score of six and above indicated adherent to ART.
111 Independent variables included individual factors such as age, education level, gender, family size,
112 employment status, and marital status. They were adopted from the Demographic Health Survey
113 (DHS) questionnaire (17). Age was collected as number of years lived. The educational status of
114 the participants was categorized as completed primary school, post-primary school training,
115 secondary ordinary level, secondary advanced level, post-secondary advanced level training,
116 college, or university. Gender was categorized as female and male. Family size was determined
117 by the number of family members residing in the household. Current marital status was categorized
118 as single, married, cohabiting, divorced, separated, or widowed. Employment status was
119 determined by asking participants to indicate whether they were self-employed, government-
120 employed, government temporary or contract employees, or private permanent or contract
121 employees.
122 The tools for assessing drug-related factors was adopted from a study conducted in Tabora
123 Tanzania (18). Participants were required to indicate the number of pills they took per day. Those
124 who reported taking more than two pills were categorized as having a high burden of pills.
125 Participants were also asked about the side effects of their ART drugs, which was categorized as
126 either experienced or not experienced side effects. Furthermore, the frequency of tabs taken per
127 day was categorized as once, twice, or more.
128 The respondents were asked if they had experienced a shortage of ART drugs in the past and if
129 they are provided with regular health education. Waiting time among study participants was
130 categorized as less than 1 hour, 1 to 5 hours and more than 5 hours and distance between the
131 respondent's home and the health facility was recorded. These factors were assessed as in a
132 previous study on assessing ART adherence among adolescents conducted in Tabora (18).
133 Wealth index was calculated using household assets obtained from a questionnaire adapted from
134 the DHS (17). Principle Component Analysis (PCA) was used to reduce dichotomized variables
135 to those leaded as factor one, which would better describe wealth index variations. Weighted
136 wealth was determined by summing up the factor loading and divided into quintiles. Food security
137 was measured using the Household Food Insecurity Access Score (HFIAS) tool which has a total
138 score of 27 and comprises 9 questions (19). The scale was used to categorize households into food
139 secure, mildly insecure, moderately insecure, and severely insecure. HFIAS had a Cronbach's
140 alpha coefficient of 0.914.

# 141 Data collection

142 Participants were interviewed in a private room at the CTC within the hospital. They were given
143 the opportunity to voluntarily consent to participate in the study and had the right to choose
144 whether to disclose information or withdraw from the study at any point during data collection.

# 145 The data collection exercise took place between 23rd January to 28thApril, 2023.

146 **Ethical consideration**
147 The study received ethical clearance from the Research and Ethics Committee of MUHAS with
148 **reference number – MUHAS-REC-01-2023-1501**. After ethical approval, the study team sought
149 authorization to carry out the study from the Tanga Town Council and the Bombo Hospital
150 administration. Before any participant could join the study, they had to go through the consenting
151 process and sign the written informed consent after it was explained to them in clear and plain
152 language. A thumb-printed informed consent was obtained in the case of illiterate participants.
153 Participants under the age of 18 were required to obtain permission from their parents or guardians.
154 The study participants' confidentiality was ensured, and no participants' names were used in the
155 questionnaire.

# 156 Data management and analysis.

157 Stata software version 15 was used for data analysis. Magnitude of adherence to ART among
158 adolescents and young adult clients living with HIV was calculated from the eight-question one-
159 month self-recall medication adherence scale tool. Participants who achieved an overall score of
160 six or above (6-8) were categorized as adherent to ART, while those with a score below six (0-5)
161 were categorized as non-adherent. Factors related to ART adherence among adolescents and young
162 adults living with HIV was determined using regression analyses. First, a bivariate analysis was
163 conducted to examine the relationship between the independent variables and adherence to ART.
164 The contingency tables were used to present the results of the association between the factors
165 (independent variables) and adherence to ART (dependent variable). Second, all independent
166 variables associated with adherence to ART with a p-value < 0.2 in bivariate analyses were entered
167 into multiple logistic regression models adjusted for age and sex. The results were reported as odds
168 ratios (OR) and adjusted odds ratios (aOR) with 95% confidence intervals. All variables with small
169 p values (P <0.2) were added one after another using forward stepwise selection until the final
170 model was generated.

# 171 Results

172 A total of 385 participants were interviewed during their attendance at the CTC clinic. **Table 1**
173 summarizes the overall demographic characteristics of the study participants and adherence. The
174 overall adherence was 65%. The participants' mean age was 17 years (SD±3.40), with a minimum
175 age of 10 years and a maximum age of 24 years. Of all participants, 270 were adolescents and 115
176 were young adults. Most of the participants were female (55%), attended secondary school (50%),
177 students (58%), single (95.5%), came from families with less than five members (55%), and lived
178 in urban settings (75%).

179

180

# 181 Table 1: Socio-demographic Characteristics of Study Participants

**Variables	Categories**

**Overall n (%)**

**Adherence n (%)**

**Non- adherence**

**n (%)**

**P-value**

|  | | 385 | 249 (65) | 136 (35) |  |
| --- | --- | --- | --- | --- | --- |
|  | 10-19 Years | 270 (70) | 170 (63) | 100 (37) | 0.281 |
| Age | 20-24 Years | 115 (30) | 79 (69) | 36 (31) |  |
|  | Female | 213 (55 | 136 (64) | 77 (36) | 0.706 |
| Sex | Male | 172 (45) | 113 (66) | 59 (34) |  |
|  | Not attended school | 34 (9) | 16 (47) | 18 (53) |  |
|  | Primary school  Secondary school | 147 (38)  194 (50) | 94 (64)  134 (69) | 53 (36)  60 (31) | 0.065 |
| Education level | University/ college | 10 (3) | 5 (50) | 5 (50) |  |
|  | Single | 368 (95.5) | 235 (64) | 133 (36) |  |
|  | Married | 6 (1.6) | 6 (100) | 0 (0) |  |
|  | Divorced | 5 (1.3) | 3 (60) | 2 (40) |  |
| Marital status | Co-habit | 5(1.3) | 4 (80) | 1 (20) | 0.343 |
|  | Widow | 1 (0.3) | 1 (100) | 0 (0) |  |
|  | Student | 222 (58) | 142 (64) | 80 (36) |  |
|  | Employed | 15 (4) | 12 (80) | 3 (20) |  |
|  | Self-employed | 66 (17) | 45 (68) | 21 (32) | 0.487 |
| Occupation | Unemployed | 82(21) | 50 (61) | 32 (39) |  |

Rural	97 (25)	55 (57)	42 (43)	0.057

Location setting	Urban	288 (75)	194 (67)	94 (33)

Family size

Less than 5 Family members

5 family members or more.

125 (32)

260 (68)

80 (64)	45 (36)

169 (65)	91 (35)

0.848

182

# 183 Factors associated with adherence to ART among participants.

184 In multivariate analysis, participants with secondary education level were 2.31 times more likely
185 to adhere to ART compared to those with no education (p =0.05). Household food insecurity was
186 also associated with ART adherence. Those from households with moderate food insecurity were
187 67% less likely to adhere to ART compared to those with severe food insecurity (p <0.001).
188 Availability of ART at the facility was also associated with adherence among adolescents and
189 young adults. For example, those who always found ART available at the facility were 4.2 times
190 more likely to adhere to the ART compared to their counterparts (p=0.02). Moreover, side effects
191 to medication were associated with ART adherence. Adolescents and young adults who
192 experienced side effects were 39% less likely to adhere to ART compared to their counterparts (p=
193 0.04).

194

195

# 196 Table 2: Factor associated with adherence to ART among adolescents and young adults

| **Variables** | **Bivariate analysis** | | | **Multivariate analysis** | | |
| --- | --- | --- | --- | --- | --- | --- |
|  | cOR | 95% CI | P | aOR | 95% CI | P |
| **Education Level** |  |  |  |  |  |  |
| Not attended school | 1.00 |  |  | 1.00 |  |  |
| Primary school | 1.99 | 0.94-4.24 | 0.07 | 1.95 | 0.86-4.41 | 0.11 |
| Secondary school | 2.51 | 1.20-5.26 | 0.02 | 2.31 | 1.02-5.23 | **0.05** |
| University/ college | 1.13 | 0.27-4.61 | 0.87 | 0.85 | 0.18-3.94 | 0.83 |
| **Food insecurity** |  |  |  |  |  |  |
| Food secure | 1.00 |  |  | 1.00 |  |  |
| Mild insecure | 0.76 | 0.45-1.27 | 0.30 | 0.76 | 1.42-1.36 | 0.35 |
| Moderate insecure | 0.31 | 0.17-0.56 | 0.00 | 0.33 | 0.16-0.66 | **0.00** |
| Severe insecure | 0.30 | 0.12-0.72 | 0.01 | 0.40 | 0.14-1.13 | 0.08 |
| **Wealth index** |  |  |  |  |  |  |
| Lowest | 1.00 |  |  | 1.00 |  |  |
| Lower | 1.96 | 1.01-3.77 | 0.05 | 0.92 | 0.40-2.10 | 0.84 |
| Middle | 1.88 | 0.98-3.60 | 0.06 | 0.96 | 0.46-2.00 | 0.91 |
| Higher | 1.67 | 0.88-3.19 | 0.12 | 1.12 | 0.54-2.33 | 0.75 |
| Highest | 2.12 | 1.10-4.10 | 0.03 | 1.42 | 0.69-2.96 | 0.34 |
| **Availability of ART** |  |  |  |  |  |  |
| No | 1.00 |  |  | 1.00 |  |  |
| Yes | 4.72 | 1.63-13.71 | 0.00 | 4.20 | 1.29-13.72 | **0.02** |

| **Waiting time to pick**  **ART** |  |  |  |  |  |  |
| --- | --- | --- | --- | --- | --- | --- |
| Less than 1 hour | 1.00 |  |  | 1.00 |  |  |
| Between 1-5 hours | 0.58 | 0.40-0.91 | 0.02 | 0.71 | 0.43-1.18 | 0.18 |
| More than 5 hours | 0.42 | 0.22-0.80 | 0.01 | 0.64 | 0.31-1.31 | 0.22 |
| **Side effect of ART** |  |  |  |  |  |  |
| No | 1.00 |  |  | 1.00 |  |  |
| Yes | 0.45 | 0.29-0.70 | 0.00 | 0.61 | 0.38-0.98 | **0.04** |
| **Frequency of daily**  **dose** |  |  |  |  |  |  |
| Once | 1.00 |  |  | 1.00 |  |  |
| Twice | 0.38 | 0.15-0.96 | 0.04 | 0.48 | 0.17-1.32 | 0.15 |

197 Key: cOR: crude Odds ratio, aOR: Adjusted odds ratio, CI: Confidence interval

198

# 199 Discussion

200 The study found that 35.3% of adolescents and young adult clients were non-adherent to ART.
201 Factors associated with adherence included household food insecurity, low level of education,
202 consistent availability of ART, and ART side effects. Addressing such unprecedented care and
203 treatment challenge for HIV in this population calls for efforts to understand and mitigate these
204 risk factors in Tanga region as in other areas.
205 More than one third of adolescents and young adults on treatment were not adherent to life-saving
206 ART. This surpasses the five percent threshold set by the World Health Organization (19).
207 Comparable findings from a separate study in Dar es salaam, Tanzania reported 40% (20), and
208 37% (20). Similar levels were found in Mwanza (37.5%) despite using viral and pill count methods
209 (21). Non-adherence levels to ART in some sub-Saharan African countries ranges 13-20%, which
210 are significantly lower compared to findings in our study and the rest of the regions in Tanzania.
211 The differencess could be owing to methods of data extraction methods risking under estimation
212 (22,23).
213 Household food insecurity was associated with ART adherence, as participants with food security
214 were more likely to adhere to ART compared to those facing various degrees of food insecurity.
215 This correlation aligns with findings in Tanzania, highlighting food insecurity as a barrier for
216 young populations living with HIV in middle- and low-income countries, impeding optimal ART
217 adherence (24). Furthermore, a narrative review across Kenya, Uganda, South Africa, and other
218 developed countries underscored the connection between food insecurity and poor adherence to
219 ART, associating it with adverse HIV health outcomes such as lower CD4 cell counts and higher
220 HIV viral loads in clients (25).
221 Adherence to ART increased with higher education levels in this study, except for those with
222 college level education. Evidence suggests that adolescents and young adults with no formal
223 education were more likely to be non-adherent compared to those with primary and secondary
224 education. Unexpectedly, individuals with university or college-level education demonstrated non-
225 adherence contrary to studies in Tanzania (24) and Ethiopia (26). It is expected that educated
226 populations are more likely to understand health promotion and warnings compared to their
227 counterparts. They are more likely to look for explanations and search for further information
228 beyond the counselling messages, therefore increasing the likelihood of adherence. Educated
229 individuals may also be socially demographically advantageous therefore in a better health
230 decision state.
231 Availability and access to ART is an important factor behind adherence to medication. The
232 availability of ART at healthcare facilities demonstrated a strong association with adherence
233 among HIV-positive adolescents. Similar evidence was reported in other Sub-Saharan African
234 countries as reported in a review of evidence done between 2004 and 2016 (27).
235 As expected, side effects are a limitation to adherence among people living with HIV and AIDS.
236 This was also demonstrated among adolescents and young adults in this study. A narrative review
237 conducted in sub-Saharan African countries reported that ART side effects hinder ART adherence
238 among children and young adolescents (4,23). Furthermore, a 2022 retrospective study in Dar es
239 Salaam found that ART side effects were associated with poor adherence to ART (21).
240 Furthermore, the guidelines for HIV management in adolescents links side effects and inadequate
241 adherence to ART. It highlights the importance of managing side effects effectively and providing
242 adequate support to adolescents and young adults on ART (28,29). ART counselling and
243 investigating side effects during all clinic visits may help mitigate early signs and complaints of
244 side effects.
245 The study was limited by its reliance on self-reported measures to collect data on ART adherence
246 and related factors, which introduces the potential for recall bias. To address this limitation, clear
247 and specific questions were used during data collection. Despite this limitation, this study fills data
248 void in adherence and factors associated with it among the vulnerable population in Tanga region.
249 Evidence presented in this study will strengthen efforts towards the fast-track targets.
250 In conclusion, the current study demonstrated a suboptimal ART adherence among adolescents
251 and young adults living with HIV and attending care and treatment facilities in Tanga region.
252 Specifically, more than one in three had a suboptimal adherence, a level far beyond the
253 recommended WHO recommendation that may be on track to reach the 95-95-95 elimination
254 targets. Factors such as food insecurity, lack of formal education, side effects, and ART shortages
255 were associated with lower adherence rates. Addressing this unprecedented level of ART
256 adherence, targeted interventions among adolescents and young adolescents are necessary to meet
257 the targets. These include, improved patient education and support for adolescents and young
258 adults with lower education levels; prioritizing interventions to address food insecurity especially
259 in such a region with adequate food production; ensuring consistent ART availability n care and
260 treatment facilities at all the times; and investigating, counseling and enhancing side effects
261 management by healthcare providers, with a focus on routine monitoring and proactive support to
262 improve overall adherence rates.

# 263 Acknowledgment

264 The authors express gratitude to the study participants, research assistants, as well as the districts
265 and regional administrative office, and Tanga Regional Referral Hospital for their invaluable
266 contributions that have made this study successful.
267 **Contribution:** SK conceptualized the study, prepared the first draft proposal, conducted data
268 collection, analyzed data, and prepared the first draft manuscript; NT provided guidance on the
269 proposal, supervised data collection, analysis, and edited the manuscript; and BS supervised and
270 edited proposal, supervised data collection, analysis, critically appraised the manuscript, and
271 approved for submission
272 **Conflicts of interest**: All authors declare no conflicts of interest.
273 **Source of Fund:** The study was part of thesis for MPH program and did not receive any external
274 fund.

275

276

277

278

279

280

281

282

283

284

285

286

287

288	**References**

289

290	1.	UNAIDS	(2022)	Global	HIV	statistics	fact	sheet	2022,	UNAIDS.	doi:

291	10.1177/004208168902500202.

292	2.	WHO, PAHO, Pan America Helath organization (2018) Multi-Country Cooperation

293	Strategy 2018-2024, WHO and PAHO. doi: 10.18372/2306-6407.1.16197.

294 3.	UNICEF (2021) Health Results 2020: HIV and AIDS. Available at: [www.unicef.org/health.](http://www.unicef.org/health)
295 4.	Ministry Of Health, Community Development, Gender, E. A. C. (2018) National
296 Adolescent Health And Development Strategy 2018 - 2022.

297

298 5.	Adejumo OA, Malee KM, Ryscavage P, Hunter SJ, Taiwo BO. Contemporary issues on the
299 epidemiology and antiretroviral adherence of HIV-infected adolescents in sub-Saharan
300 Africa: A narrative review. J Int AIDS Soc. 2015;18(1):1–19.
301 6.	He L, Yu B, Yu J, Xiong J, Huang Y, Xie T, et al. The impact of social capital and mental
302 health on medication adherence among older people living with HIV (PLWH). BMC Public
303 Health. 2021;21(1):1–8.
304 7.	Becker N, Poudel KC, Cordeiro LS, Sayer AG, Sibiya TE, Sibeko LN. A quantitative
305 analysis of food insecurity and other barriers associated with ART nonadherence among
306 women in rural communities of Eswatini. PLoS One. 2021;16(8 August 2021):1–16.
307 8.	Kadia BM, Takah NF, Dimala CA, Smith A. Barriers to and enablers of uptake of and
308 adherence to antiretroviral therapy in the context of integrated HIV and tuberculosis
309 treatment among adults in sub-Saharan Africa: A protocol for a systematic literature review.
310 BMJ Open. 2019;9(10):1–7.
311 9.	Kioko MT, Pertet AM. Factors contributing to antiretroviral drug adherence among adults
312 living with HIV or AIDS in a Kenyan rural community. African J Prim Heal Care Fam Med.
313 2017;9(1):1–7.
314 10.	Meloni ST, Agaba P, Chang CA, Yiltok E, Oguche S, Ejeliogu E, et al. PLOS ONE
315 Longitudinal evaluation of adherence , retention , and transition patterns of adolescents
316 living with HIV in Nigeria. J Am Med Assoc. 2020;1–16.
317 11.	Kim MH, Mazenga AC, Yu X, Ahmed S, Paul ME, Kazembe PN, et al. High self-reported
318 non-adherence to antiretroviral therapy amongst adolescents living with HIV in Malawi:
319 Barriers and associated factors. J Int AIDS Soc. 2017;20(1):1–12.
320 12.	UNFPA Tanzania. Fact Sheet : Young people and HIV and AIDS : Taking action to realize
321 the	90-90-90	targets.	Fact	sheet	[Internet].	2019;	Available	from:
322 https://tanzania.unfpa.org/en/publications/fact-sheet-young-people-and-hiv-and-aids-
323 taking-action-realize-90-90-90-targets
324 13	Palladium (2018) Adolescent HIV in Tanzania - Results from an Application of the
325 Pediatric HIV Transition Model, U.S. Agency for International Development.
326 14	Amour MA, Shayo GA, Matee MM, Machumi L, Rugarabamu A, Aris EA, et al. Predictors
327 of mortality among adolescents and young adults living with HIV on antiretroviral therapy
328 in Dar es Salaam, Tanzania: a retrospective cohort study. J Int AIDS Soc. 2022;25(2).
329 15	Barker C, Bowsky S, Dutta A. HIV trends among adolescents and young adults, ages 15-
330 24,	in	23	high-burden	countries	[Internet].	2016.	Available	from:
331 <http://programme.aids2016.org/Abstract/Abstract/3566>
332 16	Hansana V, Sanchaisuriya P, Durham J, Sychareun V, Chaleunvong K, Boonyaleepun S, et
333 al. Adherence to antiretroviral therapy (ART) among people living with HIV (PLHIV): A
334 cross-sectional survey to measure in Lao PDR. BMC Public Health. 2013;13(1).
335 17	Moon SJ, Lee WY, Hwang JS, Hong YP, Morisky DE. Accuracy of a screening tool for
336 medication adherence: A systematic review and meta-analysis of the Morisky Medication
337 Adherence Scale-8. PLoS One. 2017;12(11):1–18.
338 18 National Bureau of Statistics. Tanzania Demorgraphic and Health Survey Indicator Survey
339 (TDHS-MIS) 2015-2016. Vol. 1, Tanzania Mainland], Ministry of Health (MoH)
340 [Zanzibar], National Bureau of Statistics (NBS). 2016. p. 1–630.
341 19	Charles, G. (2020) Factors Associated With Antiretroviral Therapy Adherence among
342 adolescent living with HIV – a case of Kigoma region.

343

344 20	Milward de Azevedo Meiners MM, Araújo Cruz I, de Toledo MI. Adherence to
345 antiretroviral therapy and viral suppression: Analysis of three periods between 2011 and
346 2017 at an HIV-AIDS center. Front Pharmacol. 2023;14(March):1–8.
347 21	Mussa FM, Massawe HP, Bhalloo H, Moledina S, Assenga E. Magnitude and associated
348 factors of antiretroviral therapy adherence among children attending HIV care and treatment
349 clinics in Dar es Salaam, Tanzania. PLoS One [Internet]. 2022;17(9 September):1–19.
350 Available from: <http://dx.doi.org/10.1371/journal.pone.0275420>
351 22	Amour M, Sangeda RZ, Kidenya B, Balandya E, Mmbaga BT, Machumi L, et al. Adherence
352 to Antiretroviral Therapy by Medication Possession Ratio and Virological Suppression
353 among Adolescents and Young Adults Living with HIV in Dar es Salaam, Tanzania. Trop
354 Med Infect Dis. 2022;7(4).
355 23	Martelli G, Antonucci R, Mukurasi A, Zepherine H, Nöstlinger C. Adherence to
356 antiretroviral treatment among children and adolescents in Tanzania: Comparison between
357 pill count and viral load outcomes in a rural context of Mwanza region. PLoS One.
358 2019;14(3):1–15.
359 24	Lee E, Kim J, Bang JH, Lee JY. Suboptimal medication adherence in young women living
360 with HIV infection: a Korean nationwide claims study. AIDS Care - Psychol Socio-Medical
361 Asp	AIDS/HIV	[Internet].	2021;33(7):938–42.	Available	from:
362 https://doi.org/10.1080/09540121.2020.1844863
363 25	Gemechu GB. Children ’ s Adherence to Antiretroviral Therapy and Associated Factors :
364 Multi-center Cross-sectional Study. Res squareesearch Sq. 2022;1–15.
365 26	Young S, Wheeler AC, McCoy SI, Weiser SD. A Review of the Role of Food Insecurity in
366 Adherence to Care and Treatment Among Adult and Pediatric Populations Living with HIV
367 and AIDS. AIDS Behav. 2014;18:505–15.

368

369 27	Arage G, Tessema GA, Kassa H. Adherence to antiretroviral therapy and its associated
370 factors among children at South Wollo Zone Hospitals, Northeast Ethiopia: A cross-
371 sectional study. BMC Public Health. 2014;14(1).

| 372 28 | Ammon N, Mason S, Corkery JM. Factors impacting antiretroviral therapy adherence |
| --- | --- |
| 373 | among human immunodeficiency virus–positive adolescents in Sub-Saharan Africa: a |
| 374 | systematic review. Public Health [Internet]. 2018;157(0):20–31. Available from: |
| 375 | https://doi.org/10.1016/j.puhe.2017.12.010 |
| 376 29 | Abrams E, Ammann A, Anderson M, Bernstein L, Baker C. Guidelines for the use of |
| 377 | antiretroviral agents in pediatric HIV Infection January 7, 2000. HIV Clin Trials. |
| 378 | 2000;1(3):58–99. 30 |
