# Supplementary material for: Factors associated with adherence to antiretroviral therapy among HIV-positive adolescents and young adult patients attending HIV care and treatment clinic at Bombo Hospital in Tanga region-Tanzania
Source: PLoS One. 2025 Jan 3;20(1):e0316188. doi: 10.1371/journal.pone.0316188 (PMC11698315; doi:10.1371/journal.pone.0316188)
Supplement: Attachment — Submitted filename: EDITORS AND REVIEWERS COMMENTS MATRIX 15-05-2024_SK.docx [file pone.0316188.s003.docx]

**EDITORS AND REVIEWER'S COMMENTS**

**Journal Requirements:**

| **No.** | **Comment** | **Response** | **Page** |
| --- | --- | --- | --- |
| 1. | Please ensure that your manuscript meets PLOS ONE's style requirements, including those for file naming. The PLOS ONE style templates can be found at  [https://journals.plos.org/plosone/s/file?id=wjVg/PLOSOne_formatting_sample_main_body.pdf](https://eur03.safelinks.protection.outlook.com/?url=https%3A%2F%2Fjournals.plos.org%2Fplosone%2Fs%2Ffile%3Fid%3DwjVg%2FPLOSOne_formatting_sample_main_body.pdf&data=05%7C02%7Cn.tesha.1%40research.gla.ac.uk%7C9a374df1f1164a48b0fe08dc21891d9d%7C6e725c29763a4f5081f22e254f0133c8%7C1%7C0%7C638422120291463308%7CUnknown%7CTWFpbGZsb3d8eyJWIjoiMC4wLjAwMDAiLCJQIjoiV2luMzIiLCJBTiI6Ik1haWwiLCJXVCI6Mn0%3D%7C0%7C%7C%7C&sdata=aXhK8x5ZhwtXsHONqTQD2OVblJq9J7ZTKQcupUI2VaU%3D&reserved=0) and  [https://journals.plos.org/plosone/s/file?id=ba62/PLOSOne_formatting_sample_title_authors_affiliations.pdf](https://eur03.safelinks.protection.outlook.com/?url=https%3A%2F%2Fjournals.plos.org%2Fplosone%2Fs%2Ffile%3Fid%3Dba62%2FPLOSOne_formatting_sample_title_authors_affiliations.pdf&data=05%7C02%7Cn.tesha.1%40research.gla.ac.uk%7C9a374df1f1164a48b0fe08dc21891d9d%7C6e725c29763a4f5081f22e254f0133c8%7C1%7C0%7C638422120291468383%7CUnknown%7CTWFpbGZsb3d8eyJWIjoiMC4wLjAwMDAiLCJQIjoiV2luMzIiLCJBTiI6Ik1haWwiLCJXVCI6Mn0%3D%7C0%7C%7C%7C&sdata=%2BWOOD535TQ6HhdWiD%2F6oHMwx1yGctkKca0D3u2VkXqw%3D&reserved=0) | Thank you for the comments and time taken to review our manuscript. We have addressed all comments on a point-by-point basis as follows in this table and reflected in the revised manuscript. | Throughout |
| 2. | We suggest you thoroughly copyedit your manuscript for language usage, spelling, and grammar. If you do not know anyone who can help you do this, you may wish to consider employing a professional scientific editing service. | Thank you for the comment and suggestion. We have undertaken a thorough copyedit of the manuscript and worked on the language, grammar, and flow. | Throughout |
| 3. | The name of the colleague or the details of the professional service that edited your manuscript | Prof. Bruno Sunguya is a prolific academic writer and research leader at the Muhimbili University of Health and Allied Sciences. | N/A |
| 4. | A copy of your manuscript showing your changes by either highlighting them or using track changes (uploaded as a *supporting information* file)  A clean copy of the edited manuscript (uploaded as the new *manuscript* file)” | Thank you for this comment. We have submitted the recommended documents. | Throughout |
| 5. | In the online submission form, you indicated that "Data will be available upon request. The authors will contact the ethical board to abide with the country policy on sharing data internationally."  All PLOS journals now require all data underlying the findings described in their manuscript to be freely available to other researchers, either 1. In a public repository, 2. Within the manuscript itself, or 3. Uploaded as supplementary information. This policy applies to all data except where public deposition would breach compliance with the protocol approved by your research ethics board. If your data cannot be made publicly available for ethical or legal reasons (e.g., public availability would compromise patient privacy), please explain your reasons on resubmission and your exemption request will be escalated for approval. | Thank you for this comment and recommendation.  Data requests and sharing is allowed by the national laws. However, the entity that requests research data must comply with the National regulations that govern the National Health and Research Committee (NatHREC). This can be done by filling the Data Sharing Agreement under this URL chrome-extension://efaidnbmnnnibpcajpcglclefindmkaj/[https://nimr.or.tz/wp-content/uploads/2022/01/Data_Transfer_Agreement_for_Researchers.pdf](https://eur03.safelinks.protection.outlook.com/?url=https%3A%2F%2Fnimr.or.tz%2Fwp-content%2Fuploads%2F2022%2F01%2FData_Transfer_Agreement_for_Researchers.pdf&data=05%7C02%7Cn.tesha.1%40research.gla.ac.uk%7Cab9ab8d79bab4e8b453b08dc8d3a0cc5%7C6e725c29763a4f5081f22e254f0133c8%7C1%7C0%7C638540528097833191%7CUnknown%7CTWFpbGZsb3d8eyJWIjoiMC4wLjAwMDAiLCJQIjoiV2luMzIiLCJBTiI6Ik1haWwiLCJXVCI6Mn0%3D%7C0%7C%7C%7C&sdata=FVSqli91aYPwG6jykZ5MiqyvOf9t%2BzGd3Hio%2Bm08D5Q%3D&reserved=0)  Owing to the sensitivity of the subject matter, the ethical committee did not allow the data to be stored in another external repository. Authors believe in open data science and adherence to national laws. | N/A |
| 6. | We note you have included a table to which you do not refer in the text of your manuscript. Please ensure that you refer to Table 2 in your text; if accepted, production will need this reference to link the reader to Table | Thank you for the comment. We have revised the manuscript and referenced Table 2. | 12 |
| **Reviewer One** | | | |
|  | **Tittle** |  |  |
| 1 | Please reason out why you adhered to this age group. | Thank you for reviewing our manuscript and providing feedback. We have detailed the rationale for selecting the age group in the Study Population section of our revised manuscript. This has been included because adolescents aged 15 - 19 and young adults aged 19 - 24 in Tanzania have a disproportionately high number of new HIV infections, with higher mortality rates among them and the number of those living with HIV in this group is on rising. | Page 5 |
|  | **Abstract** |  |  |
|  | **Background** |  |  |
| 2 | Why couldn't you consider the magnitude of poor ART drug adherence throughout the world or if it is a public concern, rather than describing ART strength? | Thank you for your comment. We have rectified the issue and now acknowledge the importance of ART drug adherence, considering it as a significant public health concern. Globally, ART adherence rates are 77% for adults and 57% for children. In sub-Saharan Africa, only 62.3% of adolescents report ART adherence. | 2 |
| 3 | The readers should know what you mean by adherence to ART and its importance in adolescence and young adulthood. | Thank you for this comment, we defined Adherence to HIV treatment regimen as taking of all the prescribed ART pills at the correct time, in the correct doses, and in the right manner. | 2 |
| 4 | Please try to state your abstract by what is Adherence to ART, its magnitude, cause/risk factor, the consequence of poor adherence, and conclude with your objective. | Thank you for your input. We have incorporated risk factors, consequences, and concluded with the main objective accordingly. | 2 |
|  | **Methodology** |  |  |
| 5 | Needs major revision please clearly describe here the statistical software used for data entering and the way you generate statistically significant variables to your outcome variable. | Thank you for your feedback. We have addressed it by including a description of the data entering software and adding p-values as a means used to declare statistically significant results. | 2 |
|  | **Introduction** |  |  |
| 6 | Please focus on Adherence to ART drug broadly (your objective is to assess the level of Adherence to ART drug) | Thank you for your feedback. We have made the changes by introducing the global target of achieving three 95s and linking it to statistics on ART adherence among adolescents and young adults compared to all people living with HIV. We have also retained the statistics on adherence to ART in sub-Saharan Africa. | 4 |
|  | **Methods** |  |  |
| 7 | Better to write study design and setting in one paragraph rather than separately | Thank you for your comment. We have incorporated it into a single paragraph as suggested | 5 and 6 |
| 8 | Study area; must talk about the number and location of facilities in the study area and number of facilities providing ART service. But you are talking here about the prevalence of HIV, viral load which is absolutely not the right place to write | Thank you for your feedback. We have removed the information on HIV prevalence and viral load. Instead, we have included the number of health facilities that provide HIV/AIDS care and treatment in the Tanga region as suggested | 5 and 6 |
| 9 | How many referral hospitals are there in Tanga city? | Thank you for this comment, there is only one referral hospital in Tanga region where the study took place. | 5 |
| 10 | What do you think of those health centers providing ART services and adolescent and young adults attending ART treatment at health centers of Tanga City | Thank you for your input. We have acknowledged restriction to only a referral hospital as a the limitation of our study. | 16 |
| 11 | Study populations; What is the importance of stating the finding of others here? It's unclear, and needs major revisions. | Thank you for your comment. We have addressed by removing the cited references and also updated the study population details. The updated details ar the population of HIV-positive adolescents aged 15 - 19 years and young adults aged 19 - 24 years, registered at Bombo CTC and on ART for at least 6 months. Excluded were those too ill to participate. | 6 |
| 12 | Sample size; detailed sample size calculations are needed considering estimated prevalence of Adherence to ART drugs and factors significantly associated with Adherence to ART drugs | Thanks for your comment. The sample size was calculated using the Cochran's formula sample size formula from C. R. Kothari Research methodology book 2nd Edition.  N = Z^2^ × P (1-P)  e^2^  Z is the level of confidence (1.96), p is the proportion of reported adherence to ARVs among young adults (50%), and e is the absolute margin of error (5%). Therefore, the required sample size was 385. | 7 |
| 13 | Data processing and management: Initially, the process in this part is unclear. How do you code, recode and compute the scale/items and dichotomized in-to Adherent and Not adherent? | Thank you for your feedback. We have updated the manuscript to include the following details: Adherence to ART was assessed as the dependent variable using the Morisky Medication Adherence Scale (MMAS), a validated one-month self-recall scale consisting of eight response items. A score of six or above indicated adherence, while a score below six indicated non-adherence. | 7, 8 and 10 |
| 14 | What is your baseline criteria to categorize in-to six or above and below? | Thank you for your comment. We have categorized adherence to ART based on the Morisky Medication Adherence Scale (MMAS), an internationally validated tool widely used to measure adherence to chronic diseases worldwide. This is now reflected in the manuscript. | 7, 8 and 10 |
| 15 | Are you interested in logistic regression for only independent variables? What about for dependent variables? | Thank you for this comment. In our analysis regression was used to find the relationship between adherence to ART and its determinants. The dependent variable was adherence and independent variable were the factors. In the model building we assessed the relationship between adherence and each factor separately (simple logistic regression). All factors that had P < 0.2 were entered into the multiple logistic regression. | 10 |
| 16 | Is it bivariable or bivariate/multivariable or multivariate? | Thank you for this comment, this has been changed to simple logistic regression analysis and multiple logistic regression to reflect when the model had only two variables and when it had more than two variables respectively. | 10 |
| 17 | What did you mean by the results were reported as odds ratio and adjusted odds ratio? | Thank you for this comment, the Odds ratio were the results from simple logistic and adjusted odds ratio were the results after including confounding (multiple regression analysis). | 10 |
| 18 | Still you are adhered to p<0.2, how did you declare statistically significant variables? | Thank you for this comment, we have made correction as it was a typo error, significance was declared when the correction made p<0.05. | 10 |
|  | **Results** |  |  |
| 19 | Totally your result part needs major revisions |  |  |
| 20 | Why are you writing the dependent variable in the sociodemographic characteristics? | Thank you for your feedback. We have addressed it by removing the dependent and independent variables. Only descriptive statistics of the sociodemographic characteristics are retained. | 11 & 12 |
| 21 | Is it the right place to see the association and p-value in the sociodemographic characteristics? | Thank you for your feedback. We have removed the association in the sociodemographic characteristics | 11 & 12 |
| 22 | Age classifications? | Thank you for your feedback. We have incorporated the age classification according to the specifications outlined by the World Health Organization (WHO), where individuals aged 10 to 19 years are categorized as adolescents, and those aged 20 to 24 years are classified as young adults. This classification adheres to global standards. | 11 |
| 23 | Totally your regression table is WRONG |  |  |
| **Reviewer # 2** | | | |
|  | **General** |  |  |
| 1 | The paper needs language and grammar editing. | Edited by Prof. Bruno Sunguya |  |
|  | **Abstract** |  |  |
| 2 | Remove unnecessary abbreviations in the abstract | Thank you for your feedback. We have removed all unnecessary abbreviations from the abstract.. | 2 |
| 3 | Your conclusion needs revision; it would be better if you added your recommendation rather than repeating your findings | Thank you for your feedback. We have made the following change as more than one-third of adolescents and young adults adhere to treatment. Efforts targeting this population are crucial to achieve the three 95% adherence target. | 3 |
|  | **Methods** |  |  |
| 4 | It would be better if you added the health services related to the problem to the study area | Thank you for your suggestion. We have considered it and incorporated health services related to the problem into the study area. | 6 |
| 5 | Why have you only considered a single institute as your study area? | Thank you for this comment, and we have been added as a limitation of the study accordingly | 16 |
| 6 | Sample size determination The author reported that he used 84% of the adherence level of the previous study, but it is not clear how it could be 384. Please clarify the procedure you used to calculate the sample size because I cannot obtain the number you mentioned using the single population proportion formula. In your description, you presented 385 data points. How could it be??? | Thanks for your comment. The sample size was calculated using the Cochran's formula sample size formula from Research methodology book 2nd Edition.  N = Z^2^ × P (1-P)                 e^2^  Z is the level of confidence (1.96), p is the proportion of reported adherence to ARVs among young adults (50%), and e is the absolute margin of error (5%). Therefore, the required sample size was 385. | 7 |
| 7 | Data Processing and Analysis  The author said bivariable and multivariate analyses were carried out to check for significant variables. But you did not test the confounding factors, and you also didn’t check the goodness of fit of the model. Why? | Thank you for this comment. In our analysis regression was used to find the relationship between adherence to ART and its determinants. The dependent variable was adherence and independent variables were the factors. In the model building we assessed the relationship between adherence and each factor separately (simple logistic regression). All factors that had P < 0.2 were entered into the multiple logistic regression. | 10 |
|  | **Results** |  |  |
| 8 | From the beginning, what is the significance of this study? What makes it different from the previous studies conducted in different areas of the country? Let me hear your new findings. Look at your descriptive part; it should be modified to be more attractive to readers | Thank you for this comment, this study contributes valuable insights into the current status of adherence to antiretroviral therapy among adolescents and young adults, a group that has been relatively under-researched in Tanzania. By focusing on this population, the study offers up-to-date information regarding ART adherence, addressing a gap in existing research. Additionally, the study's key findings, such as the correlation between food insecurity and ART adherence, shed light on the pressing need for improved food security measures within this group. | 14 |
|  | **Discussion** |  |  |
| 9 | Your justification is not strong enough; there are many studies conducted on your problem that are expected to be discussed with those findings. Rewrite it Generally, the discussion part needs strong justifications | Thank you for this comment. We have rewritten it and added recent studies. | 13- 16 |
|  | Reference |  |  |
| 10 | Some of your references are outdated; please revise them | Thank you for this comment, we have removed the old reference and the information it had on the issue of viral load and replace it with the reference of the health facilities that serve the Tanga region. | 6 & 21 |
| **Reviewer Three** | | | |
|  | **General** |  |  |
| 1 | Strengths of the manuscript | Acknowledged with thanks |  |
|  | **Weakness** |  |  |
| 2 | The Authors did not use any figures to show concepts, procedures, or results | Thank you for this comment, we have answered our objective by using descriptive statistics and regression analysis. | 11-14 |
|  | **Major issues** |  |  |
|  | **Sample size determination** |  |  |
| 3 | You didn't calculate a sample size for your secondary objective (for factors) to check and take the large sample size | Thank you for this comment, we calculated our sample based on the dependent variable adherence. We had many independent variables so we did not use them in our sample calculation. The sample size was calculated using the Cochran's formula sample size formula from Research methodology book 2nd Edition.  N = Z^2^ × P (1-P)                 e^2^  Z is the level of confidence (1.96), p is the proportion of reported adherence to ARVs among young adults (50%), and e is the absolute margin of error (5%). Therefore, the required sample size was 385. | 7 |
| 4 | The prevalence of adherence to ART was 84%. Therefore, you were supposed to use only 2% of margin of error to increase the number of your sample size | Thank you for this comment, we adjusted for non-response by adding a 50% of the calculated sample to increase the number of the sample size. | 7 |
|  | **Minor issues** |  |  |
|  | **In the abstract part** |  |  |
| 5 | Please write the exposing and preventing factors separately. It seems a repetition of the main result. Therefore, mention only the names of variables significantly associated with your dependent variable. (Like A,B,C,and D were significantly associated.) | Thank you for your feedback. We have re-written the factors separately. | 2 |
| 6 | Better to write 'moderate food insecurity with (AOR 0.33 (95%CI 0.16-0.66)) and those experiencing ART side effects with AOR 0.61, 95%CI 0.38-0.98) were LESS LIKELY to adhere….. X and Y (CI, AOR) were MORE LIKELY to | Thank you for this comment, we have re-written as per the comment. | 2 |
| 7 | Please review the punctuation marks | Thank you for this comment, the punctuation has been reviewed and corrected accordingly. | 2 and 3 |
|  | **Under introduction** |  |  |
| 8 | Please, add more information about the solutions that have been tried in terms of successes and failures with only one paragraph. | Thank you for this comment, we have added information on the solutions which have been used in the past as follows: Recent systematic review on the intervention to increase uptake of HIV treatment found that counselling, incentives, mobile phone short message service (SMS), peer delivered behavioral intervention, community ART delivery, electronic adherence service monitoring device, lay health worker lead group intervention, and food assistance to be effective. These interventions are either effective individually or when combined. However, it further showed that incentives (either financial or in kind), SMS and electronic adherence monitoring device interventions have varying statistically significant or non-significant effects in different settings. | 5 |
| 9 | Add gaps in the previous studies from what you reviewed. | Thank you for this comment, the gap has been added as follows: the evidence on the factors for adherence has been documented in other setting, but for Tanzania the level of adherence to ART its factors was lacking. | 5 |
| 10 | Sample size determination |  |  |
|  | You did not mention the number of your total population. If your population is less than ten thousand, you could use a correction formula | Thank you for this comment, we adjusted for non-response by adding a 50% of the calculated sample to increase the number of the sample size. | 6 and 7 |
|  | Under result |  |  |
| 11 | Be consistent while you write your paper. For instance, the prevalence of adherence to ART in the abstract part and the result part is not consistent (64.7 and 65%). | Thank you for this comment, we have changed and consistently use 64.7%. | 11 |
| 12 | Table 2 Factors associated with adherence to ART  A frequency table should be included that shows the percentage of the adherent and non-adherent groups to see and check classification bias. Even it helps to check the Odds ratio manually | Thank you for this comment, we have added the frequency and their percentage | 12 & 13 |
|  | **Under discussion part** |  |  |
| 13 | Your manuscript says that the differences could be owing to methods of data extraction methods risking under-estimation. It will be better if you clarify the tool differences (extraction methods) between yours and the others in a positive manner | Thank you for this comment, we have added the information on other tools used to measuring ART adherence by other comparable studies as follows: The variations in ART adherence rates could stem from differences in data collection methods and the diverse approaches used to measure adherence. For instance, methodologies such as pill count and pharmacy refill systems are commonly utilized but may carry the risk of overestimating adherence. Conversely, recall methods might underestimate adherence levels. Therefore, this study opted to employ an internationally validated method known for its high reliability. | 14 |
|  | **Reference related** |  |  |
| 14 | There are some outdated references like Abrams E, Ammann A, Anderson M, Bernstein L, Baker C. Guidelines for the use of antiretroviral agents in pediatric HIV Infection January 7, 2000. HIV Clin Trials. 2000;1(3):58–99. 30 | Thank you for this comment, we have removed the old reference and the information it had on the issue of viral load and replace it with the reference of the health facilities that serve the Tanga region. | 6 & 21 |
