# Supplementary material for: Factors associated with adherence to antiretroviral therapy among HIV-positive adolescents and young adult patients attending HIV care and treatment clinic at Bombo Hospital in Tanga region-Tanzania
Source: PLoS One. 2025 Jan 3;20(1):e0316188. doi: 10.1371/journal.pone.0316188 (PMC11698315; doi:10.1371/journal.pone.0316188)
Supplement: Attachment — Submitted filename: EDITORS AND REVIEWERS COMMENTS MATRIX 16-08-2024 - .doc [file pone.0316188.s005.doc]

**EDITORS AND REVIEWER'S COMMENTS**

**Journal Requirements:**

| **No.** | **Comment** | **Response** | **Page** |
| --- | --- | --- | --- |
| 1. | The manuscript has some grammatical issue. Needs to be revised | Thank you for the comment and suggestion. We have undertaken a thorough copyedit of the manuscript and worked on the language, grammar, and flow. | Throughout |
| **Reviewer** | | | |
|  | **Tittle** |  |  |
|  | **Abstract** |  |  |
| 2 | Background should be organized and restated well. It seems too bulky and not concisely. | Thank you for reviewing our manuscript and providing valuable feedback. We have revised the background section to enhance its clarity and coherence. | Page 2 |
|  | **Methodology** |  |  |
| 3 | Data from google forms not clear. Did you use for data entry or what? Entered or exported? | Thank you for your comment. We have revised the explanation for clarity. Data were collected using Google Forms, extracted as a Microsoft Excel file, and imported into STATA software for data management and analysis. | Page 3 |
|  | **Introduction** |  |  |
| 4 | Paragraph 2, Lack coherence and flow of ideas, better to rewrite. See all the parts accordingly for better understanding. | Thank you for your comment. We have rewritten paragraph 2 for clarity and logical flow. | Page 4 & 5 |
|  | **Methods** |  |  |
| 5 | Sample size calculation issue, not adequately addressed.  Where did you get 50%? It seems, you have used this proportion due to lack of similar study. Have you been considered the prevalence in Dares salaam which is 63%, or other recent study finding even it resulted under estimate? | Thank you for this comment. While the prevalence of ART adherence in Dar es Salaam is reported at 63%, we opted not to use this figure due to significant differences in culture, economics, and community dynamics between Dar es Salaam and the Tanga region. The HIV landscape varies significantly across the country. In addition, the indicators reported in Dar es salaam vary significantly compared to that of Tanga. These include HIV prevalence, incidence rate, all the 95-95-95 indicators specifically, in addition to cultural and beliefs of the people in general. Therefore, we used a 50% prevalence estimate for our sample size calculation. This decision is supported by the limitation of similar studies in these regions, which necessitates a conservative estimate to avoid over- or underestimation. Furthermore, using a 50% prevalence is a standard practice in sample size calculations when prior data is limited, as it maximizes the required sample size, ensuring sufficient statistical power. This approach is appropriate for new studies and provides a robust baseline for future research in regions with similar community characteristics.  The sample size was calculated using the sample size formula from C. R. Kothari's Research Methodology Book 2nd Edition.  N = Z2 × P (1-P)  e2  where Z represents the level of confidence (1.96), P is the proportion of reported adherence to ARVs among young adults in Tanga, Tanzania (50%), and e is the absolute margin of error (5%). The total sample size was 384. | Page 6 |
|  |  |  |  |
| 6 | For the second objectives also? Needs to be justified clearly. | Thank you for your comment. We have provided justifications for each of the variables. | Page 7 & 8 |
