# Supplementary material for: Factors associated with adherence to antiretroviral therapy among HIV-positive adolescents and young adult patients attending HIV care and treatment clinic at Bombo Hospital in Tanga region-Tanzania
Source: PLoS One. 2025 Jan 3;20(1):e0316188. doi: 10.1371/journal.pone.0316188 (PMC11698315; doi:10.1371/journal.pone.0316188)
Supplement: Attachment — Submitted filename: Reviewer comments to author.docx [file pone.0316188.s004.docx]

PLOS ONE

Factors associated with adherence to antiretroviral therapy among HIV-positive

adolescents and young adult patients attending clinic at Bombo Hospital in Tanga

region-Tanzania.

**Minor revision**

Dear author thank you for a significant change you made to the manuscript. I kindly recommend you to make a minor revision and need clarification on some issue points.

- The manuscript has some grammatical issue. Needs to be revised
- Abstract: Background should be organized and restated well. It seems too bulky and not concisely.

Method: Data from google forms not clear. Did you use for data entry or what? Entered or exported?

- Introduction: Paragraph 2, Lack coherence and flow of ideas, better to rewrite. See all the parts accordingly for better understanding.
- Methodology: Sample size calculation issue, not adequately addressed.

Where did you get 50%? It seems, you have used this proportion due to lack of similar study. Have you been considered the prevalence in Dares salaam which is 63%, or other recent study finding even it resulted under estimate?

- For the second objectives also? Needs to be justified clearly.

Regards.
